# First Report of a Case with Needle Track Sinus after Aspiration Biopsy of a Benign Thyroid Nodule Resulted in an Unexpected Postoperative Complication

**DOI:** 10.4061/2010/759109

**Published:** 2010-03-07

**Authors:** Lutfi Dogan, Niyazi Karaman, Ali Kucuk, Cihangir Ozaslan, Can Atalay, Sait Celebioglu

**Affiliations:** Department of General Surgery, Ankara Oncology Training and Research Hospital, 06340 Ankara, Turkey

## Abstract

Fine needle aspiration biopsy is the most feasible, safe, and accurate diagnostic tool for thyroid nodule diagnosis. The development of a sinus tract between thyroid gland and the skin through needle tract after fine needle aspiration biopsy is an extremely uncommon phenomenon. In this paper, a 71-year-old man presenting with a swelling and discharge on the anterior neck wall was reported. Similar complaints were present 15 to 20 days after fine needle aspiration biopsy of thyroid gland four years ago. Bilateral total thyroidectomy was performed considering a thyroid malignancy infiltrating the skin. Histopathologic examination confirmed a sinus tract between the thyroid gland and skin and thyroid nodule was benign in nature. It must be kept in mind that inflammatory reactions might also occur after fine needle aspiration biopsy of benign thyroid nodules. In patients with needle biopsy-related inflammation, surgery may be delayed until the inflammation subsides.

## 1. Introduction

Multinodular goitre (MNG) is the most common endocrine disease. The incidence of palpable thyroid nodules is 3%–7%. In addition, more than 50% of the population have thyroid nodules detected at ultrasonographic (USG) examination [1]. Noninvasive methods such as radionucleide thyroid scintigraphy and thyroid USG have been used for the diagnosis of thyroid nodules for decades. Fine needle aspiration biopsy (FNAB) guided by USG or palpation is the most feasible diagnostic tool for thyroid nodule evaluation due to low false positive (≤2%) and false negative rates (≤4%) [2]. FNAB is accepted as a simple and feasible procedure in the recent years [3]. However, like other invasive procedures, FNAB may also lead to various complications. In this paper, we report a case with thyrocutaneous sinus formation after diagnostic FNAB.

## 2. Case

Seventy-one years old male patient was admitted to the hospital with the complaints of a swelling and discharge on the anterior neck wall. These complaints were present for 10 days. In the medical history of the patient, similar complaints were present 15 to 20 days after FNAB of thyroid gland performed four years ago, and these complaints had spontaneously resolved with antibiotics within one month. At that time, no surgical intervention related to this complaint was performed.

**Figure 1:**
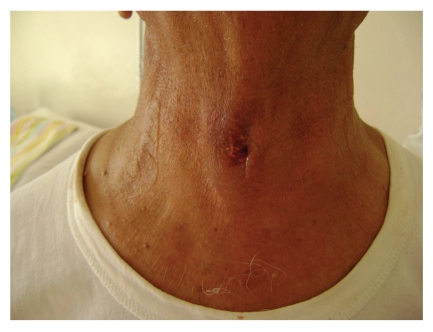
Sinus opening with moderately swollen and hyperemic skin around it.

Physical examination of the patient revealed a sinus opening with seropurulant discharge located at midline, two cm caudal to thyroid cartilage and the tissues surrounding the sinus opening were moderately swollen and hyperemic (Figure 1). There was also a palpable nodule four cm in size with circumscribed margins located in the left lobe of thyroid gland. There were no palpable lymph nodes in the cervical region. Other systemic examinations were normal. White blood cell count, neutrophil, eosinophil, C reactive protein, sedimentation rate, liver and thyroid function tests, and antithyroid antibodies were all in normal range. In cervical USG, there was a nodule 46 × 32 mm in size with multiple calcifications and mixed echogenities in the left thyroid lobe and it was pushing trachea away from the midline. At thyroid scintigraphy, the nodule was hypoactive and almost filling the whole lobe of the thyroid gland. In addition to this nodule, there was asymmetrical thickening of tracheal wall on the left side starting from the level of epiglottis and obliterating lateral recess at the level of Eustachian tube in computerized tomography (CT).

**Figure 2:**
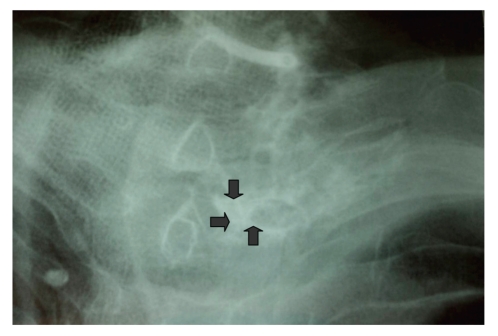
Fistulography showing contrast material passing to thyroid gland and confined to the left lobe (three arrows).

Swab culture of the wound revealed no microorganisms. The contrast material given through the opening of the sinus passed neither to trachea nor to esophagus and the sinus was completely intrathyroidal (Figure 2). USG-guided FNAB with 25 Gauge needle was performed for the nodule located in the left lobe of thyroid gland. The cells, with prominent nucleus and elongated cytoplasm, fragmented connective tissue elements and polimorphonuclear cells were noted at cytological evaluation. The result of the FNAB was suspicious for malignancy. PET-CT was also performed with the suspicion of undifferentiated thyroid malignancy infiltrating the skin. Moderate 18-FDG uptake seen on the left side of the neck from epiglottic level to cephalad direction was interpreted as an inflammatory process. However, 18-FDG uptake by thyroid nodule was reported to be suspicious for malignancy. With these radiological and clinical findings, bilateral total thyroidectomy was performed. Methylene-blue dye was injected through the opening of sinus tract at the beginning of surgery. Although the dye was totally intrathyroidal, the sinus tractus, prethyroidal muscles, and the skin were resected with the specimen. The sinus tract was continous with the left lobe of thyroid gland and there were severe inflammatory adhesions around the ligament of Berry at this region. The dissection of the left lobe was quite difficult, and central lymph node dissection was also performed. 

Esophagocutaneous fistula developed at the third postoperative day. Oral food intake was stopped and the fistula spontaneously resolved with parenteral nutrition. The histopathological examination of the specimen revealed that the sinus tract was formed by spindle cell proliferation and the tract was fixed to prethyroideal muscles. Spindle cell proliferation and inflammatory cell infiltration within vascular structures were responsible for adhesions between thyroid gland and prethyroideal muscles. The spindle cells had myofibroblastic features with no nuclear atypia. Hurtle cell changes confined within the bounderies of the nodule were noted outside the fistulous tract. These areas were stained with CD56, CK19, and HBME1 and not with Gal3 and P53. The other lobe and isthmus were made up of normal thyroidal tissue. All nine lymph nodes dissected from central region were reactive in nature. The diagnosis of the nodule was benign and the patient is still followed with no complications.

## 3. Discussion

FNAB is used for rapid cytologic diagnosis of not only thyroid nodules but also soft tissue masses of head and neck area with low morbidity rate. Neck pain and discomfort are the most frequently reported complaints after this procedure. It is thought that these complaints are related to small hematomas occuring during needle enterance to the gland. Most hematomas are self-limiting and do not cause serious problems [4]. For many years, it has been a subject of discussion that FNAB of thyroid cancers has a risk of seeding of malignant cells through needle track. In the literature, there are a few reports on subcutaneous nodule formation due to malignant cell implantation after FNAB and this was accepted as a rare cause of thyroid cancer recurrence [5]. Besides, other rare complications have also been reported as case reports. These are intrathyroidal massive hemorrhage and upper respiratory tract congestion [6], nodule infarction [7], recurrent laryngeal nerve palsy [8], tracheal injury related hemoptysis, and pleural injury resulting in pneumothorax [9].

To the best of our knowledge, there are two case reports about sinus formation through the needle track in the English literature. The diagnosis of the first reported case was in a patient with Riedel thyroiditis and it is well known that fistulas and sinuses can be seen in this disease after surgery or percutaneous manipulations [9]. The second case was reported from India and the reason for sinus formation was needle-track seeding of papillary thyroid cancer cells [10]. In the case presented here, we did not suspect about Riedel thyroiditis because of localized proliferation in the lesion. The diagnosis was not inflammatory fibrous tumor, due to ALK and desmin negativity and sinus tract formation. Anaplastic carcinoma was also excluded, since there were no atypical and CK positive cells between fibroblasts and the history of the disease was quite long. Hurthle cell carcinoma was also excluded due to its invasive properties and p53 negativity. This is the first report about thyro-cutaneous sinus formation after FNAB of a benign thyroid nodule.

The vasculature of thyroid gland increases and expands in cases of noduler goiter with hyperthyroidism. Thyroid nodules also have some aberrant vessels and these vessels are relatively thin-walled. It is thought that these vessels running in tissue planes between nodules and surrounding thyroid lobules rupture due to penetrating trauma caused by FNAB [11]. This bleeding results in hematoma formation. Intrathyroidal hemorrhage appears to be commonly associated with multinodular goiters that have thin-walled, abundant vessels [12]. Many small hematomas heal with formation of minimal fibrosis and scar tissue, but rarely a hematoma can cause widespread inflammation by increasing vascular and fibroblastic proliferative activity [4]. This inflammation or hemorrhagic necrosis can cause formation of a sinus or fistula by fixation of a large nodule to the prethyroid muscles.

The case presented here was operated with the suspicion of thyroid malignancy when there was active inflammation in the tissues. Minor tissue traumas during the dissection of areas with severe inflammation were thought to be the reason for esophago-cutaneous fistula development. In practice, 25 Gauge needles are recommended to obtain sufficient tissue sample during thyroid needle biopsy [13]. However, especially for larger solitary nodules, it may be advisable to use thinner needles to reduce local trauma. For the patients with needle biopsy related inflammation, surgery may be delayed until the inflammation subsides.

## References

[1] Ezzat S, Sarti DA, Cain DR, Braunstein GD (1994). Thyroid incidentalomas: prevalence by palpation and ultrasonography. *Archives of Internal Medicine*.

[2] Agrawal S (1995). Diagnostic accuracy and role of fine needle aspiration cytology in management of thyroid nodules. *Journal of Surgical Oncology*.

[3] Baloch ZW, LiVolsi VA (2004). Fine-needle aspiration of thyroid nodules: past, present, and future. *Endocrine Practice*.

[4] Tsang K, Duggan MA (1992). Vascular proliferation of the thyroid: a complication of fine-needle aspiration. *Archives of Pathology and Laboratory Medicine*.

[5] Karwowski JK, Nowels KW, McDougall IR, Weigel RJ (2002). Needle track seeding of papillary thyroid carcinoma from fine needle aspiration biopsy: a case report. *Acta Cytologica*.

[6] Roh J-L (2006). Intrathyroid hemorrhage and acute upper airway obstruction after fine needle aspiration of the thyroid gland. *Laryngoscope*.

[7] Jayaram G, Aggarwal S (1989). Infarction of thyroid nodule: a rare complication following fine needle aspiration. *Acta Cytologica*.

[8] Hulin SJ, Harris KP (2006). Thyroid fine needle cytology complicated by recurrent laryngeal nerve palsy and unnecessary radical surgery. *Journal of Laryngology and Otology*.

[9] Block MA, Miller JM, Kini SR (1980). The potential impact of needle biopsy on surgery for thyroid nodules. *World Journal of Surgery*.

[10] Basu A, Sistla SC, Siddaraju N, Verma SK, Iyengar KR, Jagdish S (2008). Needle tract sinus following aspiration biopsy of papillary thyroid carcinoma: a case report. *Acta Cytologica*.

[11] Terry WI (1992). Radium emanations in exophthalmic goiter: blood vessels of adenomas of the thyroid. *The Journal of the American Medical Association*.

[12] Gauger PG, Guinea AI, Reeve TS, Delbridge LW (1999). The spectrum of emergency admissions for thyroidectomy. *American Journal of Emergency Medicine*.

[13] Falk SA (1997). *Thyroid Disease: Endocrinology, Surgery, Nuclear Medicine and Radiotherapy*.

